# *Notch3* is an asymmetric gene and a modifier of heart looping defects in *Nodal* mouse mutants

**DOI:** 10.1371/journal.pbio.3002598

**Published:** 2025-03-31

**Authors:** Tobias Holm Bønnelykke, Marie-Amandine Chabry, Emeline Perthame, Gregor Dombrowsky, Felix Berger, Sven Dittrich, Marc-Phillip Hitz, Audrey Desgrange, Sigolène M. Meilhac

**Affiliations:** 1 Université Paris Cité, Imagine—Institut Pasteur Unit of Heart Morphogenesis , INSERM UMR1163, Paris, France; 2 Sorbonne Université, Collège Doctoral, Paris, France; 3 Institut Pasteur, Université Paris Cité, Bioinformatics and Biostatistics Hub, Paris, France; 4 Department for Medical Genetics, University of Oldenburg, Oldenburg, Germany; 5 Department of Congenital Heart Disease, Pediatric Cardiology Deutsches Herzzentrum der Charité, Charité Universitätsmedizin Berlin, Berlin, Germany; 6 Department of Pediatric Cardiology, University Hospital Erlangen, Friedrich-Alexander-University of Erlangen-Nürnberg, Erlangen, Germany; 7 German Center for Cardiovascular Research (DZHK), Kiel, Germany; University of Pennsylvania School of Medicine, UNITED STATES OF AMERICA

## Abstract

The TGFβ secreted factor NODAL is a major left determinant required for the asymmetric morphogenesis of visceral organs, including the heart. Yet, when this signaling is absent, shape asymmetry, for example of the embryonic heart loop, is not fully abrogated, indicating that there are other factors regulating left–right patterning. Here, we used a tailored transcriptomic approach to screen for genes asymmetrically expressed in the field of heart progenitors. We thus identify *Notch3* as a novel left-enriched gene and validate, by quantitative in situ hybridization, its transient asymmetry in the lateral plate mesoderm and node crown, overlapping with *Nodal*. In mutant embryos, we analyzed the regulatory hierarchy and demonstrate that *Nodal* in the lateral plate mesoderm amplifies *Notch3* asymmetric expression. The function of *Notch3* was uncovered in an allelic series of mutants. In single neonate mutants, we observe that *Notch3* is required with partial penetrance for ventricle thickness, septation and aortic valve, in addition to its known role in coronary arteries. In compound mutants, we reveal that *Notch3* acts as a genetic modifier of heart looping direction and shape defects in *Nodal* mutants. Whereas *Notch3* was previously mainly associated with the CADASIL syndrome, our observations in the mouse and a human cohort support a novel role in congenital heart defects and laterality defects.

## Abstract 2

The secreted factor NODAL is a left determinant required for the asymmetric morphogenesis of organs, including the heart, but other contributing factors remain unknown. This study identifies Notch3 as a novel asymmetric factor in the lateral plate mesoderm that acts as a genetic modifier of NODAL to regulate heart morphogenesis.

## Introduction

Left–right patterning of the embryo is essential for the formation of visceral organs [1,2]. Anomalies in this process lead to the heterotaxy syndrome, a severe condition including complex congenital heart defects which determine the prognosis of patients [3]. The mechanisms of symmetry-breaking are now well established in the mouse [4]. Chirality of tubulin underlies the rotational movement of motile cilia in the pit of the left–right organizer, also referred to as the node. This generates a leftward fluid flow sensed by crown cells, resulting in the asymmetric expression of the left determinant NODAL in the lateral plate mesoderm. Genetic alterations to the formation of the node, ciliogenesis or NODAL signaling are associated with heterotaxy in both mice [2,5–8] and humans [9,10]. NODAL signaling is then sensed by organ precursors to modulate morphogenesis, as shown in the intestine [11] and heart [12]. In the absence of *Nodal*, some features are symmetrical (spleen, bronchi, lung lobes, atrial appendages), but many others (e.g., heart looping, stomach, gut) retain some level of asymmetry, although abnormal [6,12–15]. This indicates that *Nodal* is not always required to initiate asymmetry and that there are other factors of asymmetry in addition to NODAL signaling.

The heart is the first organ to undergo asymmetric morphogenesis in the embryo. This manifests as a rightward looping of the heart tube primordium, at E8.5 in the mouse [16]. Heart looping is not only a direction, but also a 3D shape, which conditions the establishment of the asymmetric heart function in a double blood circuit. We have previously reconstructed the dynamics of this process and defined morphological and quantitative staging criteria from E8.5c to E8.5j, including the repositioning of ventricles from cranio-caudal to left–right, and the left displacement of the venous pole [17]. We have shown that heart looping is primarily a buckling mechanism, when the heart tube elongates between fixed poles [17]. We found that *Nodal* is not required for buckling and thus to initiate looping. In contrast, *Nodal* biases buckling, by amplifying and coordinating asymmetries independently at the two poles of the heart tube [12]. Thus, in the absence of *Nodal* in the lateral plate mesoderm, asymmetries are reduced and the helical shape of the looped heart tube is abnormal. Because two asymmetries, at the arterial and venous poles, have a randomized orientation, *Nodal* mutants display 4 classes of abnormal heart loop. We observed that NODAL signaling is transient, before the formation of the heart tube (E8.5c-d), in precursor cells of the heart tube poles. Cardiac precursor cells are progressively incorporated into the heart tube, as a main driver of its elongation. Patterning of the field of cardiac precursors has been studied in terms of molecular profiling [18] and fate [19]. However, the left–right patterning of cardiac precursors has remained centered on the *Nodal* pathway.

Other players of asymmetry have been identified, including bone morphogenetic protein (BMP) [20–22], hedgehog (HH) [23], NOTCH [24–26] and WNT [27–29] signaling. Few of these are reported to be asymmetric: *Wnt3* in the left side of the node [28], and BMP signaling in the right lateral plate mesoderm [21]. They were all shown to be required for *Nodal* asymmetry. Thus, the factors which can provide asymmetry besides *Nodal* have remained enigmatic.

NOTCH signaling is an example of a pathway required both for left–right asymmetry and for heart morphogenesis [30], but playing multiple roles. Among the four paralogues, *Notch2* is expressed in the node, and *Notch1* around the node in the caudal epiblast and presomitic mesoderm [24,31]. *Notch1;Notch2* double mutants disrupt heart looping asymmetry, in keeping with a role in the left–right organizer [25]. NOTCH1 and the transcriptional NOTCH effector RBPJ are required in the heart field for cardiomyocyte differentiation [32–34]. *Notch1* is later expressed in the endocardium, where it is important to regulate myocardial trabeculation [35] and valve formation [36,37]. *Notch3* mutant mice are viable and fertile [38], but display defects in the maturation of the smooth muscle wall of coronary arteries [39]. *Notch3* is a broad marker of pericytes and controls the formation of smooth muscles in non-cardiac arteries and intestinal lacteals [40–42]. Gain-of-function of NOTCH3 in CADASIL syndrome is responsible for smooth muscle degeneration in the brain [40,43,44]. *Notch3* is also involved in the regulation of quiescence and stemness of neural, skeletal muscle and mammary stem cells [45–48]. Truncation of NOTCH3 in the Lateral Meningocele Syndrome, is associated with cardiac defects, including aortic anomalies and septal defects [49]. Finally, *Notch4* is expressed together with *Notch1* in endothelial cells to regulate vascular remodeling [50]. Thus *Notch1/2* but not *Notch3/4* have been previously associated with left–right patterning, at the level of the left–right organizer.

To uncover asymmetric factors in organ specific precursors, rather than the left–right organizer, recent transcriptomic screens were performed on micro-dissected tissues excluding the node [51,52]. Whereas NODAL signaling is transient [12,53], these screens were based on pooled embryos, which limits their resolution. The asymmetric candidate genes that have been identified have not yet been analyzed for functional significance.

Here, we focused on asymmetric expression in the field of heart progenitors, at the time of heart looping. We compared paired left/right samples in single embryos to avoid confounding individual or stage variations. Our screen and quantitative in situ validations reveal *Notch3* as a novel gene enriched in the left lateral plate mesoderm. *Notch3* is co-expressed with *Nodal*, although with a slight time-delay and partial spatial overlap. In mutants, we show that *Notch3* asymmetry is amplified by NODAL signaling. Single *Notch3* mutants do not develop heterotaxy, but show ventricle, aortic valve, septation and coronary artery defects after birth. Analysis of compound mutants indicate that *Notch3* is a genetic modifier of heart looping defects in *Nodal* mutants. Our analysis in the mouse is supported by observations in a patient cohort, identifying novel rare *NOTCH3* variants associated with cardiac defects similar to that found in Lateral Meningocele Syndrome or in heterotaxy. Overall, we identify not only a novel asymmetric gene, but also a novel player in the left–right patterning of the lateral plate mesoderm, relevant to congenital heart defects.

## Results

### Identification of *Notch3* as a novel asymmetric marker

**Fig 1:**
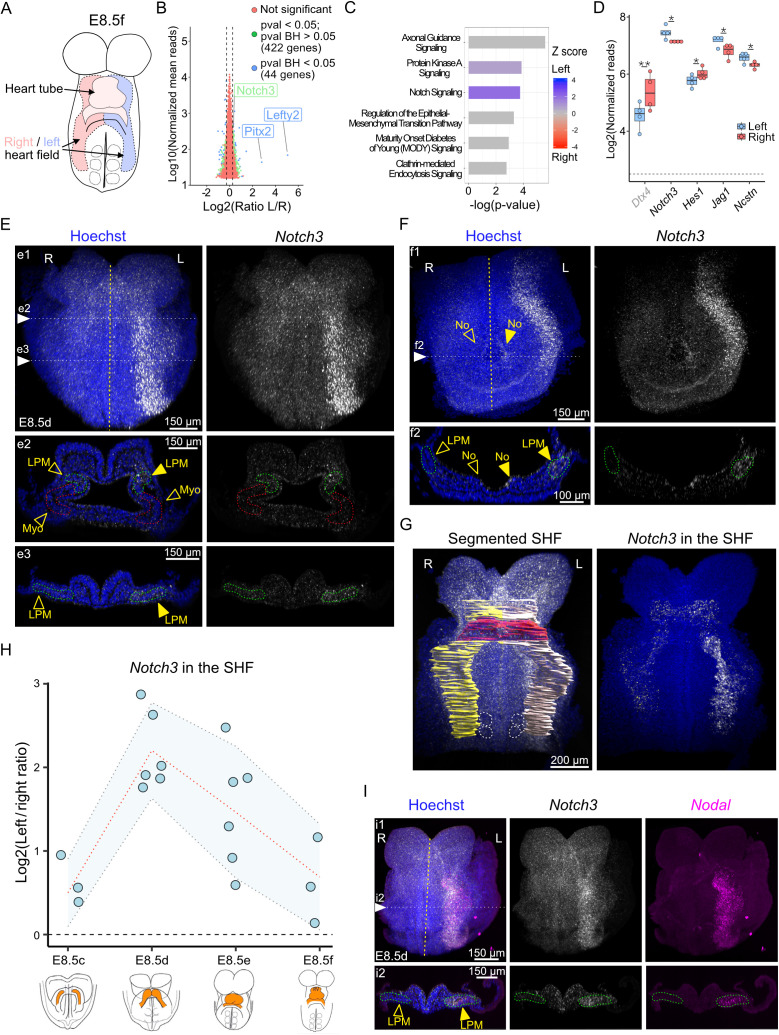
RNA-sequencing of paired samples identifies a left-sided enrichment of *Notch3* at E8.5. (**A**) Outline of the left (light blue) and right (light red) heart field micro-dissected for RNA-sequencing at the indicated stage of mouse heart looping. (**B**) MA-plot representing relative gene expression between paired left and right heart field at E8.5f. Non-significant differential expression is represented in red, differential expression in green (*p*-value < 0.05), and blue (Benjamini–Hochberg (BH) corrected *p*-value < 0.05, LimmaVoom, *n* = 4 embryos). (**C**) Ingenuity Pathway Analysis on the gene list shown in S1 Table ordered according to significance and color-coded for the activation state (*z*-score: blue, active pathway on the left; red, on the right). (**D**) Normalized read counts of genes involved in the *Notch* pathway in the left (blue) and right (red) heart field. The dotted line indicates the threshold of background expression. Whisker plots show the median, 25th- and 75th quartiles (boxes), and the extreme data points (whiskers). *p-value < 0.05, **Benjamini–Hochberg corrected *p*-value < 0.05 (LimmaVoom, *n* = 4). (**E**, **F**) Expression of *Notch3* (white) detected by whole mount RNAscope ISH in E8.5d wild-type embryos, shown in frontal views (e1, cranial part of the embryo, f1, caudal part of the embryo) and transverse sections (e2–e3, f2, at the levels indicated in e1–f1) (*n* = 6). Filled and empty arrowheads point to high and low expression, respectively. (**G**) Segmentation of the cardiac region in 3D images, to quantify gene expression in the left (white) or right (yellow) heart field. The heart tube is colored in red. Somites are outlined by white dotted lines. Expression of *Notch3* within the segmented second heart field is extracted in the right panel. (**H**) Quantification of normalized *Notch3* asymmetric expression in the heart field at sequential stages. The means are shown on a red dotted line and standard deviations are in blue (*n* = 3 at E8.5c, 6 at E8.5d, 6 at E8.5e, 3 at E8.5f). (**I**) *Notch3* (white) and *Nodal* (magenta) co-expression detected by double whole mount RNAscope ISH in E8.5d wild-type embryos, shown in a frontal view (i1) and transverse section (i2) (*n* = 5). The midline is indicated by a yellow dotted line. HT, heart tube; L, left; LPM, lateral plate mesoderm (green dotted outline); Myo, myocardium (red dotted outline); No, node; R, right; SHF, second heart field. See also S1 Video and S1 Data for the underlying data.

To screen for left–right asymmetric gene expression in cardiac cells, aside from the *Nodal* pathway, we used a transcriptomic approach in the mouse embryo. To reduce confounding factors, the approach was targeted in space and time and left/right comparisons were conducted on single rather than pooled embryos. We focused on the heart field, given that it is a tissue which can be bisected at the midline and is patterned by NODAL signaling [12]. In contrast, the heart tube changes its position during looping [17] and has a left–right origin difficult to dissect [19]. We have previously shown that NODAL ignaling is transient in heart precursors [12], highlighting the importance of specifically staging embryos. We focused on the E8.5f stage, when we have detected morphological asymmetry at the arterial pole of the heart tube [17]. Thus, we micro-dissected the heart field of E8.5f wild-type embryos (Fig 1A and S1A–S1D Fig), in the dorsal pericardial wall and down to the second somite, in agreement with landmarks of fate mapping experiments [19] and profiling of single cardiac cells [18]. Bulk RNA sequencing of paired left and right samples was compared to identify differential asymmetric expression in the heart field. As expected, targets of NODAL signaling, *Pitx2* and *Lefty2*, were significantly enriched on the left side (Fig 1B). A total set of 597 genes were predicted to be differentially expressed (S1 Table). Ingenuity Pathway analysis showed NOTCH signaling as the strongest asymmetric pathway and left-sided (Fig 1C). We controlled the transcriptomic levels of individual genes associated with NOTCH in this differential analysis, and their cardiac expression in a published single cell transcriptomic dataset at the same stage [18]: whereas *Dtx4* was found to be expressed only in ectodermal cells (S1E Fig), *Notch3*, *Hes1*, *Jag1*, *Ncstn* were identified as asymmetric candidate genes in the heart field (Fig 1D).

We selected *Notch3* for further validation, because it is the gene with highest expression, it encodes a receptor and transcription co-factor central to NOTCH signaling. This *Notch* paralogue has not previously been associated with left–right patterning. We mapped *Notch3* expression in whole mount E8.5 wild-type embryos using sensitive RNAscope in situ hybridization (ISH). *Notch3* was detected throughout the lateral plate mesoderm, as well as in the crown of the node, with higher expression on the left side (Fig 1E and 1F). Although *Notch3* expression in the node is low, we found it significantly enriched on the left side (S2 Fig). *Notch3* was not expressed in the myocardium of the forming cardiac tube. We then assessed the dynamics of *Notch3* expression within the heart field (Fig 1G). Our quantification shows that *Notch3* is transiently asymmetric, reaching 4.5-fold left-sided enrichment in the heart field at E8.5d, a stage when the myocardium bulges out to form a tube (Fig 1H). Although *Nodal* asymmetry starts one stage earlier (E8.5c, [12]), *Notch3* and *Nodal* overlapped in the left lateral plate mesoderm at E8.5d (Fig 1I, S1 Video), and were found co-expressed in single cells (S1F Fig). *Notch3* exhibits a distinct expression pattern relative to its paralogues and stands out as the only one with asymmetry: in agreement with previous reports [31], *Notch1* was detected in the endothelium and somites, *Notch2* in the somites, neural floorplate. In addition, we found low *Notch2* expression in the juxta-cardiac field and lateral plate mesoderm (S3 Fig). We have thus identified *Notch3* as a novel left-sided gene in the heart field.

### *Nodal* is required in the lateral plate mesoderm to amplify *Notch3* asymmetric expression

**Fig 2:**
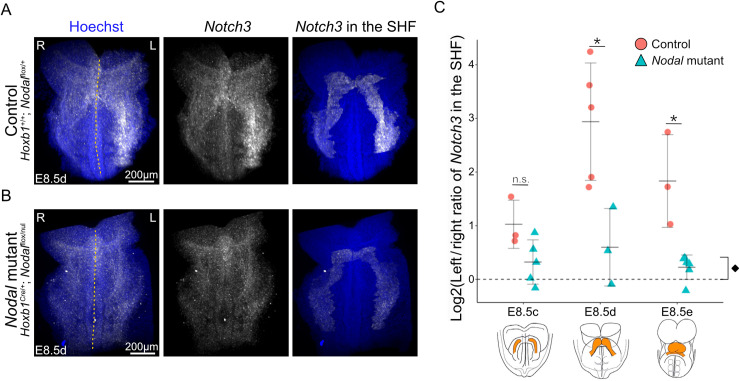
Decreased *Notch3* asymmetry in *Nodal* mutants. (**A**, **B**) Whole mount RNAscope ISH of *Notch3* in control (A) and *Nodal* mutant (B) at E8.5d in frontal views. Expression of *Notch3* within the segmented heart field is extracted in right panels. The midline of the embryo is indicated by a yellow dotted line. (**C**) Corresponding quantification of normalized *Notch3* asymmetric expression in the heart field at sequential stages in littermate controls (*n* = 3 at E8.5c, 5 at E8.5d, 3 at E8.5e) and *Nodal* mutants (*n* = 5 at E8.5c, 3 at E8.5d, 6 at E8.5e). Means and standard deviations are shown. * *p*-value < 0.05 between controls and mutants, ^♦^*p*-value < 0.05 to compare mutant levels at all stages with a symmetry hypothesis (Log2 ratio = 0) (Pairwise Mann–Whitney Wilcoxon tests, *n* = 11 controls and 14 mutants). L, left; R, right; SHF, second heart field. See also S1 Data for the underlying data.

Given that *Notch3* asymmetry overlaps with that of *Nodal*, we investigated whether *Notch3* and *Nodal* depend on each other. NOTCH signaling has been shown to directly activate *Nodal* in the node, in *Dll1* and *Rbpj* mutants [26]. In contrast, we found that *Nodal* was correctly patterned in the node and left lateral plate mesoderm of *Notch3*^*−/−*^ mutants (S4 Fig). In reverse, in mutants in which *Nodal* is inactivated in the lateral plate mesoderm but not in the node [12], *Notch3* expression was impacted (Fig 2A and 2B). Quantification of *Notch3* expression at sequential stages in the heart field shows that *Notch3* asymmetry is severely decreased in *Nodal* mutants compared to controls (Fig 2C). Yet, some significant asymmetry is still detectable. In keeping with a partial dependency of *Notch3* on *Nodal*, we observed that the expression domain of *Notch3* partially overlaps with that of *Nodal*, extending more medially and less laterally (S1G and S1H Fig). Thus, *Nodal* is required in the left lateral plate mesoderm to amplify, rather than initiate, *Notch3* asymmetric expression.

### Single *Notch3* mutants have normal heart looping, but later congenital heart defects

**Fig 3:**
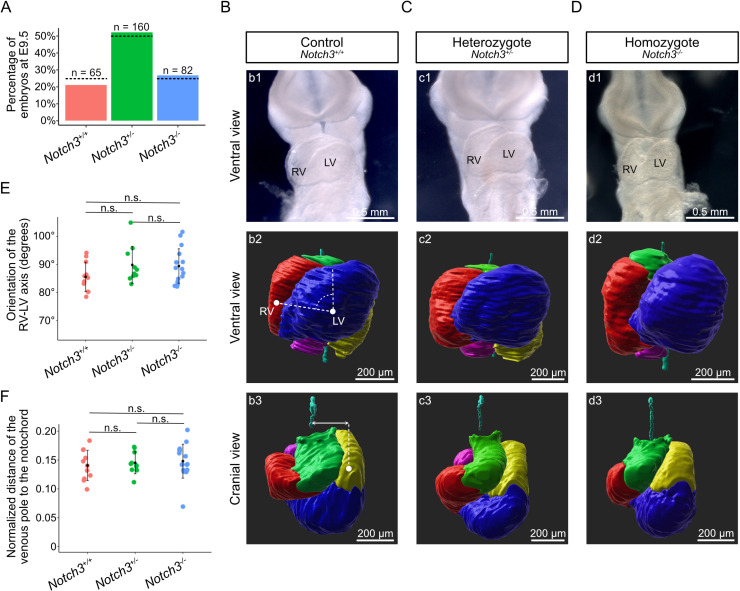
Normal heart looping in single *Notch3* mutants. (**A**) Histogram showing the percentage of genotypes recovered in E9.5 litters of *Notch3*^*+/−*^ × *Notch3*^*+/−*^ crosses. The observed frequency is not significantly different from the expected mendelian ratio (dotted lines) (*p*-value =  0.30, chi-squared test, *n* as indicated). (**B**–**D**) Brightfield ventral views (b1–d1), and 3D segmented heart tubes shown in ventral (b2–d2) and cranial (b3–d3) views, of littermate *Notch3*^*+/+*^ (B), *Notch3*^*+/−*^ (C) and *Notch3*^*−/−*^ (D) E9.5 embryos. Cardiac regions are color coded: outflow tract in green, right ventricle in red, left ventricle in blue, atrioventricular canal and left atrium in yellow, right atrium in magenta. The notochord (cyan) is used as a reference axis to align samples. (**E**) Quantification of the orientation of the RV/LV axis relative to the notochord, as schematized in b2. (**F**) Quantification of the displacement of the venous pole relative to the notochord, normalized by the tube length, as schematized in b3. Means and standard deviations are shown (Pairwise Mann–Whitney Wilcoxon tests with Benjamini–Hochberg correction, *n* = 11 *Notch3*^*+/+ *^, 10 *Notch3*^*+/−*^ and 16 *Notch3*^*−/−*^). LV, left ventricle; n.s., non-significant; RV, right ventricle. See also S1 Data for the underlying data.

We next investigated the role of *Notch3* in heart development, in mutants inactivated for it (S5 Fig). Given its asymmetric expression when the cardiac tube forms, we looked for a potential effect on the rightward looping of the heart tube. E9.5 mutant embryos were collected at mendelian ratio (Fig 3A) and no heart looping defect was detected, both qualitatively (Fig 3B–3D) and quantitatively (Fig 3E and 3F). *Notch3*^*−/−*^ mutant embryos were indistinguishable from controls, with a normal orientation of the right ventricle-left ventricle axis relative to the midline, and a normal left displacement of the venous pole.

**Fig 4:**
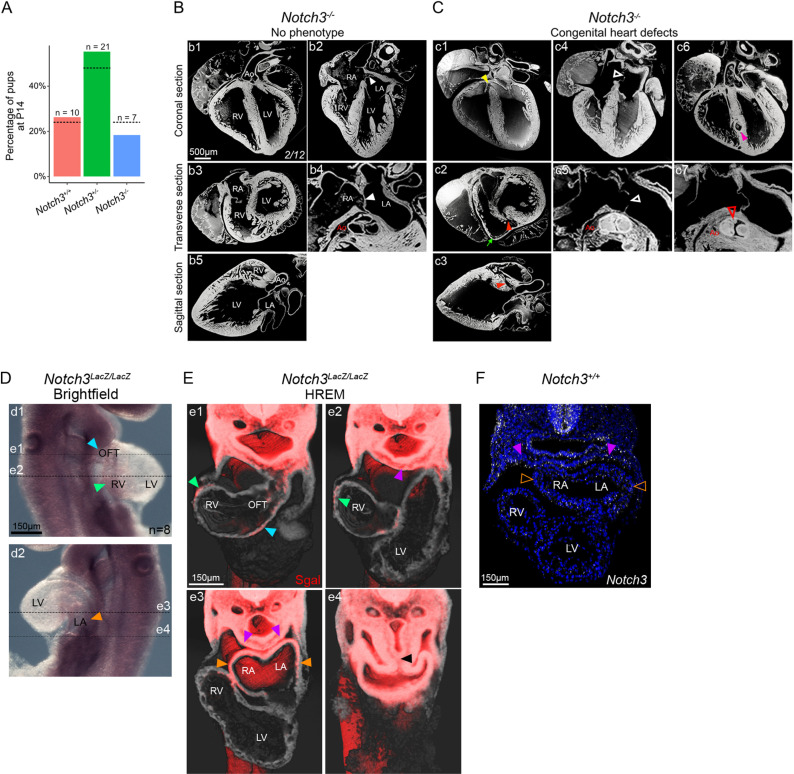
Congenital heart defects in *Notch3* mutants. (**A**) Histogram showing the percentage of genotypes recovered in P14 litters of *Notch3*^*+/−*^ × *Notch3*^*+/−*^ crosses. The observed frequency is not significantly different from the expected mendelian ratio (dotted lines) (*p*-value =  0.64, chi-squared tests, *n* as indicated). (**B**, **C**) Sections of *Notch3*^*−/−*^ mutant hearts at P0, imaged by HREM. An example of a mutant heart with no phenotype is shown (B, *n* = 2/12). The white arrowhead shows atrial septation. Defects (C) include peri-membranous ventricular septal defect (VSD) (yellow arrowhead, *n* = 1/12), muscular VSD (red arrowheads, *n* = 4/12), secundum atrial septal defect (empty white arrowhead, *n* = 2/12), dilation of the septal coronary artery (pink arrowhead, n = 1/12), hypoplastic non coronary leaflet (empty red arrowhead, *n* = 1/12) and thin RV wall (green arrow, *n* = 3/12). (**D**) Brightfield images of a *Notch3*^*lacZ/lacZ*^ embryo at E9.5, shown in right (d1) and left (d2) lateral views. Cells that have previously expressed *Notch3* are stained with Sgal. (**E**) Serial transverse sections from 3D HREM images at the levels indicated in D. Sgal staining is in red and histology in gray. The purple arrowhead indicates the dorsal pericardial wall or second heart field, and the black arrowhead the posterior dorsal mesocardium which contributes to the atrial septum. (**F**) Whole mount RNAscope ISH of *Notch3* in a wild-type embryo at E9.5, shown in a transverse section. Filled and empty arrowheads point to high and low expression, respectively. Ao, aorta; LA, left atrium (orange arrowhead); LV, left ventricle; OFT, outflow tract (blue arrowhead); RA, right atrium (orange arrowhead); RV, right ventricle (green arrowhead). See also S1 Data for the underlying data.

We then collected *Notch3* mutants after birth. In agreement with previous reports [38], they were collected at mendelian ratio (Fig 4A). Unlike *Nodal* mutants, *Notch3*^*−/−*^ mutants did not show heterotaxy, had no anomaly in the laterality of visceral organs (S6A and S6B Fig). However, they had congenital heart defects with partial penetrance (83%), including peri-membranous and muscular ventricular septal defects, thinning of the right ventricular wall, secundum atrial septal defects, hypoplasia of the non-coronary leaflet of the aortic valve, coronary artery dilation (Fig 4B and 4C). Defects of coronary arteries are in keeping with fetal expression of *Notch3* in pericytes and smooth muscle cells (S6C Fig). We performed genetic tracing to analyze the contribution of precursor cells that have expressed *Notch3* to the E9.5 heart tube. Whereas *Notch3* is mainly expressed at E9.5 in dorsal pericardial wall precursors (Fig 4F), *Notch3*^*lacZ*^-positive cells, which have previously expressed *Notch3*, were detected in the outflow tract, right ventricle and atria (Fig 4D and 4E). They were also detected in precursors of the dorsal mesenchymal protrusion which participate in atrial septation (Fig 4e4). Overall, our results show that *Notch3* is required for the outflow tract, right ventricle, atrial septation and coronary artery development. However, removal of *Notch3* alone does not uncover a role in left–right asymmetric organogenesis.

### *Notch3* is a genetic modifier of heart looping defects in *Nodal* mutants

**Fig 5:**
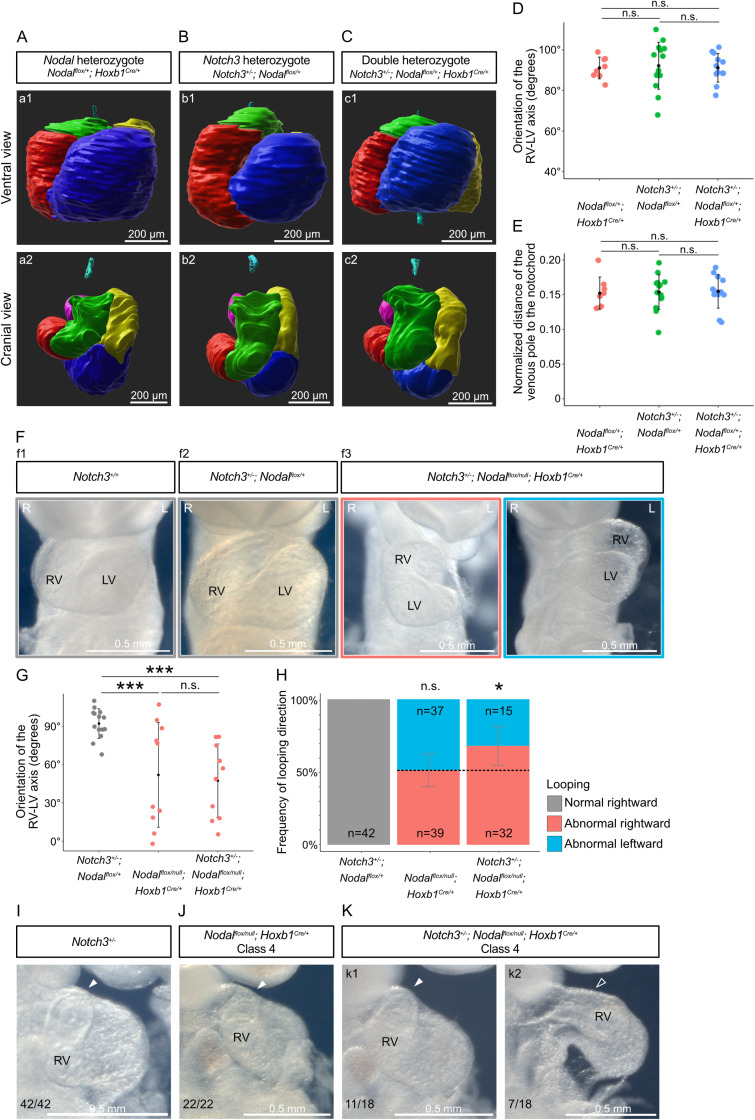
Heart looping variations in *Notch3* and *Nodal* compound mutants. (**A**–**C**) 3D segmented heart tubes shown in ventral (a1–c1) and cranial views (a2–c2) of single *Nodal* heterozygote (A), single *Notch3* heterozygote (B) and double heterozygote (C) embryos at E9.5. Cardiac regions are color coded as in Fig 3. The notochord (cyan) is used as a reference axis to align samples. (**D**) Quantification of the orientation of the RV/LV axis relative to the notochord. (**E**) Quantification of the displacement of the venous pole relative to the notochord, normalized by the tube length. Pairwise Mann–Whitney Wilcoxon tests with Benjamini–Hochberg correction (*n* = 8 *Hoxb1*^*Cre/+ *^*; Nodal*^*flox/+ *^*,* 14 *Notch3*^*+/−*^*; Nodal*^*flox/+ *^*,* 12 *Notch3*^*+/−*^*; Hoxb1*^*Cre/+ *^*; Nodal*^*flox/+*^). (**F**) Brightfield images of wild type (f1), control *Notch3* heterozygote (f2) compared to homozygote *Nodal* mutants with decreased *Notch3* (f3) at E9.5, in a ventral view. (**G**) Quantification of the orientation of the RV/LV axis relative to the notochord in rightward loops. Mann–Whitney Wilcoxon test with Benjamini–Hochberg correction (*n* = 14 *Notch3*^*+/−*^*; Nodal*^*flox/+ *^*,* 10 *Nodal*^*flox/null*^*; Hoxb1*^*Cre/+*^ (Class2 and 4)*,* 10 *Notch3*^*+/−*^*; Nodal*^*flox/null*^*; Hoxb1*^*Cre/+*^). (**H**) Quantification of the looping direction frequency in homozygote *Nodal* mutants with decreased *Notch3* (right bar), which, in contrast to *Nodal* mutants with normal *Notch3* levels (middle bar), differs from a randomized looping direction (dotted line). *p-value < 0.05 (chi-squared test with Yates’ continuity correction, *n* = 42 *Notch3*^*+/−*^*; Nodal*^*flox/+*^*; n* = 76 *Hoxb1*^*Cre/+*^*; Nodal*^*flox/null*^ and 47 *Notch3*^*+/−*^*; Hoxb1*^*Cre/+ *^*; Nodal*^*flox/null*^). (**I**–**K**) Comparison of Class 4 abnormal heart loops in homozygote *Nodal* (J), *Nodal; Notch3* compound (K) mutants compared to control *Notch3*^*+/−*^ embryos (I), seen in brightfield right-sided views*.* Filled and empty arrowheads point to curved and straight outflow tract, respectively. Means and standard deviations are shown. L, Left; LV, left ventricle; n.s., non-significant; R, Right; RV, right ventricle. See also S1 Data for the underlying data.

Since *Notch3* overlaps with and is amplified by *Nodal*, we investigated their interaction in the context of left–right asymmetry. We generated double heterozygote mutants and quantified heart looping. Compared to single heterozygotes (Fig 5A and 5B), *Notch3*^*+/−*^*; Hoxb1*^*Cre/+ *^*; Nodal*^*flox/+*^ double heterozygotes (Fig 5C) did not show any heart looping defects, including normal orientation of the right ventricle-left ventricle axis relative to the midline, and normal left displacement of the venous pole (Fig 5D and 5E). Thus, we did not detect a genetic interaction between *Notch3* and *Nodal*.

We then analyzed the role of *Notch3* in a sensitized background. We generated a large cohort of mutants, with a knock-down of *Notch3* within the context of *Nodal* inactivation in the left lateral plate mesoderm. *Notch3*^*+/−*^*; Hoxb1*^*Cre/+ *^*; Nodal*^*flox/null*^ mutants lost the randomized direction of asymmetric heart looping, typical of *Nodal* mutants (Fig 5F and 5H). Rightward looping was more frequent when *Notch3* was reduced in *Nodal* mutants. However, this did not correspond to a rescue, since all rightward mutant heart loops were abnormal, compared to controls (Fig 5G). Variation in the distribution of looping direction rather reflects a change in the laterality of the underlying asymmetry at the arterial pole. Compared to *Nodal* mutants, additional defects in the outflow tract were observed with partial penetrance. The outflow tract was straighter in 40% (7/18) of *Notch3*^*+/−*^*; Hoxb1*^*Cre/+ *^*; Nodal*^*flox/null*^ mutants within looping Class 4 (Fig 5I–5K), affecting overall the asymmetric shape of the heart tube. Outflow tract defects are classical laterality defects [12,54]. This indicates that even the less severe class of looping defects in *Nodal* mutants is exacerbated by *Notch3* knock-down. Our genetic analyses thus uncover a role for *Notch3* as a genetic modifier of heart looping defects in *Nodal* mutants.

### *NOTCH3* variants in patients with congenital heart defects

**Fig 6:**
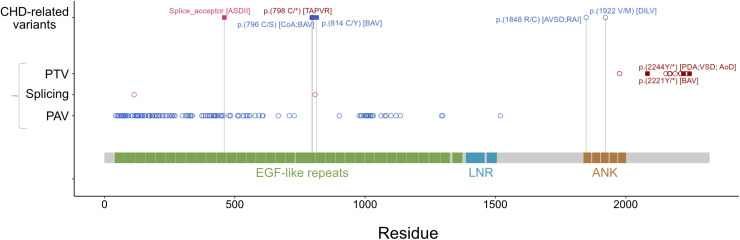
Novel *NOTCH3* variants identified in patients with congenital heart defects, compared with previously reported clinical variants. The first line shows ultrarare and likely pathogenic *NOTCH3* variants (*n* = 6) identified in a cohort of 3,907 patients with congenital heart defects. Below are 424 *NOTCH3* variants previously reported as pathogenic or likely pathogenic in ClinVar, including CADASIL cases (*n* = 127). Filled squares indicate variants associated with Lateral Meningocele syndrome in ClinVar (*n* = 3) or variants with heart defects similar to that found in patients with Lateral Meningocoele syndrome (*n* = 3). ANK, ankyrin repeat; AoD, aortic dilation; ASDII, secundum atrial septal defect; AVSD, atrioventricular septal defect; BAV, bicuspid aortic valve; CoA, coarctation of the aorta; DILV, double inlet left ventricle; LNR, LIN-12/notch repeat; PAV (blue dots), protein altering variant (missense, inframe deletion/insertion); PDA, persistent ductus arteriosus; PTV (brown dots), protein truncating variant (stop gain, frameshift); RAI, right atrial isomerism; Splicing (pink dots), canonical splice site variant; TAPVR, Total anomalous pulmonary venous return; VSD, ventricular septal defect. See also S2 Table.

We interrogated a large cohort to investigate whether *NOTCH3* could be involved in congenital heart defects in patients. We found six ultrarare *NOTCH3* variants, classified as (likely) pathogenic (PM2, according to the criteria of the American College of Medical Genetics and Genomics (ACMG), [55]) (Fig 6, S2 Table). Three patients display secundum atrial septal defect, bicuspid aortic valve or coarctation of the aorta. These defects are similar to those found in *Notch3*^*−/−*^ mouse mutants and in patients with Lateral Meningocele syndrome [49]. In a single patient, besides *NOTCH3*, we identified rare pathogenic variants in *RAF1* and *GJA5* (S2 Table). Three other patients have atrioventricular septal defect, right atrial isomerism, total anomalous pulmonary venous return, or double inlet left ventricle. These defects can be found in patients with heterotaxy [3] or mouse models of heterotaxy such as *Hoxb1*^*Cre/+ *^*; Nodal*^*flox/null*^ mutants [12]. No additional (likely) pathogenic variant in a list of plausibly associated heterotaxy genes could be identified in these patients. These observations suggest that *NOTCH3* could be involved in congenital heart defects in patients. Further work will be required to confirm variant pathogenicity and penetrance. If confirmed, human genetic variants would support the double role of *NOTCH3* shown in mice: for heart valve and septation, and for heart left–right asymmetry (heterotaxy spectrum).

## Discussion

We have identified *Notch3* as a novel left asymmetric factor. We show that it is amplified by NODAL signaling. We do not detect a genetic interaction between *Nodal* and *Notch3*, but rather show that *Notch3* acts as a genetic modifier of heart looping defects in *Nodal* mutants. Knock-down of *Notch3* changes the distribution of looping direction and exacerbates loop shape defects observed in *Nodal* mutants. Single *Notch3* mutant neonates have other cardiac defects in coronary arteries, aortic valve, transmural growth of the right ventricle, and septation. The double role of *Notch3* in heart development and left–right patterning is supported by observations of *NOTCH3* variants in patients with congenital heart defects relevant or not to heterotaxy.

Left–right asymmetry is mainly analyzed in terms of patterning of the major left determinant, *Nodal*, and its downstream pathway including *Lefty2* and *Pitx2* [4,56,57]. In the absence of *Nodal*, heterotaxy occurs with full penetrance, still organ asymmetry is not fully abrogated [6,13–15]. Heart looping, which is the first asymmetric morphogenesis, still occurs to some extent in *Nodal* mutants, producing helical heart tubes, even if abnormal [12]. This indicates that there are other factors than NODAL signaling required for heart asymmetry. BMP [20–22], HH [23], NOTCH [24–26] and WNT [27–29] signaling have been shown to be required upstream of *Nodal* for asymmetry. Transcriptomic screens have been performed to identify asymmetric genes in embryos outside the node or in cardiac precursors [51,52]. Yet, asymmetric candidate genes were either not validated or not analyzed functionally, leaving open the question of which are genuine asymmetry factors. Here, we have used a transcriptomic screen tailored spatially in the heart field, tailored temporally at the stage of the first morphological asymmetry, and tailored specifically on left–right asymmetry based on paired comparison in single embryos. This has successfully identified a novel left factor, *Notch3*. We have characterized by quantitative and whole mount sensitive ISH its dynamic spatio-temporal expression pattern relative to *Nodal*. We have also demonstrated its role in left–right asymmetry based on an allelic series of mutants. Similarly to *Nodal*, *Notch3* is transiently asymmetric, highlighting the importance of careful staging to monitor left–right patterning. In contrast to *Nodal*, *Lefty2* and *Pitx2,* which are exclusively expressed on the left of the lateral plate mesoderm, *Notch3* is also expressed on the right, with a 4.5-fold enrichment on the left side. Thus, quantitative and sensitive approaches are critical to uncover factors which have lower asymmetry levels compared to the *Nodal* pathway.

*Nodal* plays multiple roles in asymmetry. *Nodal* is well known to act as a bias, able to set the laterality of asymmetry, so that its absence leads to a randomized orientation. This is the case for the direction of heart looping [12] or the stomach position [14]. In addition, *Nodal,* upstream of *Pitx2*, acts as a regulator of identity, able to confer a left anatomical structure to the atria and lungs or to induce the formation of the spleen [14,58,59]. Finally, we had shown that *Nodal* can also act as an amplifier of asymmetry [12]. This had been detected at a morphological level, with a reduced rotation of the arterial pole during heart looping in *Nodal* mutants. We now show this at a molecular level, with the amplification of *Notch3* asymmetry by *Nodal*.

NOTCH signaling is required at different levels for left–right asymmetry. *Notch1/2* were shown previously to play a role in the formation of the left–right organizer and the induction of *Nodal* [24–26]. We now identify the role of another NOTCH paralogue, NOTCH3. Whereas *Notch1* is expressed around the node in the caudal epiblast and presomitic mesoderm, and *Notch2* in the node pit [24,31], we have detected *Notch3* in the node crown and lateral plate mesoderm. Our conditional mutants permit to dissect a role of *Notch3* in the lateral plate mesoderm for left–right asymmetry. How *Notch3* regulates heart looping direction and the curvature of the outflow tract remains an open question. *Notch3* regulates muscle differentiation and maturation in other tissues, such as the smooth muscles of arteries, coronary arteries and intestinal lacteals, or skeletal muscles, and is expressed in precursors cells such as pericytes or satellite stem cells [39–42,45]. It will be interesting to investigate whether *Notch3* in the heart field similarly regulates the differentiation and maturation of the cardiac muscle to impact heart looping. Elongation of the heart tube is important for heart looping, as a driver of a buckling mechanism [17]. Thus, asymmetries in myocardial cell differentiation, when precursor cells incorporate into the heart tube, can provide a left–right bias to orient looping. We have shown previously that *Nodal* modulates a large set of genes involved in myocardial cell differentiation, some of which like *Tnnt1* are asymmetrically expressed within the wild-type heart tube [12]. Whether *Notch3* acts similarly remains to be investigated. For later transmural growth and septation of ventricles, it is possible that a potential role of *Notch3* on muscle differentiation and maturation intervenes. However, since *Notch3* is expressed in cardiac precursors and not cardiomyocytes, that would imply a long delay from expression to phenotype. Another possibility is that ventricle growth in *Notch3* mutants is a secondary consequence of defective coronary vascularization [60].

Although *Notch3* expression is sensitive to *Nodal*, our observations support the idea that *Notch3* is not a component of the *Nodal* pathway. The expression pattern of *Notch3* differs from *Nodal*, with a right sided and more medial left sided localization. In contrast to *Pitx2* or *Lefty2* [12], *Notch3* expression is not erased in *Nodal* mutants. Functionally, we do not detect a genetic interaction between *Notch3* and *Nodal*, and show that knock-down of *Notch3* can change the phenotype of *Nodal* mutants. Thus, *Notch3* comes out as a novel genetic modifier of heart looping defects in *Nodal* mutants. The underlying mechanism is still unknown. A link between the two pathways has been detected based on the direct binding of NOTCH3 to the NODAL co-receptor TDGF1, which enhances NOTCH proteolytic maturation and sensitization to ligand-induced NOTCH signaling [61]. This suggests synergy between the NODAL and NOTCH3 pathways. Given that the disease associated with *Nodal* dysfunction, heterotaxy, has a heterogenous phenotypic spectrum, the identification of genetic modifiers of heterotaxy opens novel perspectives to fill gaps in the genetic diagnosis of 60% of patients [62].

In humans, *NOTCH3* was so far mainly associated with neural and vascular defects, such as in CADASIL, Sneddon and Lateral Meningocele syndromes [43,49,63]. Yet, patients with Lateral Meningocele syndrome also show cardiac defects, such as aortic anomalies and septal defects. The Lateral Meningocele syndrome is not associated with decreased *NOTCH3* expression, but rather with *NOTCH3* variants in exon 33, leading to protein truncation. We now observe cardiac defects typical of Lateral Meningocele syndrome in homozygote *Notch3* mouse mutants and in other patients with *NOTCH3* variants not in exon 33, but in the EGF-like repeats. We also extend the spectrum of phenotypes associated with *NOTCH3* variants, to cardiac defects typical of heterotaxy. Three of the here described heterozygote (likely) pathogenic variants in humans resemble phenotypes also observed in heterotaxy. It is currently unknown if these new variants of *NOTCH3* associated with cardiac defects behave differently than those observed in patients with CADASIL syndrome. Further studies are required on these variants individually to obtain mechanistic insight and confirm their involvement in heterotaxy. Our work in the mouse overall shows a double role of *Notch3*, during heart development and as a genetic modifier of laterality defects of the heart.

## Methods

### Experimental model and subject details

C56Bl6J mice were used as wild-type embryos. *Notch3*^*tm1Grid/tm1Grid*^ mutants (abbreviated *Notch3*^*−/−*^, [38]) were maintained in a C56Bl6J background; they are viable and fertile as homozygotes. *Nodal*^*null/+*^*; Hoxb1*^*Cre/+*^ males [12] were maintained in a mixed genetic background and crossed with *Nodal*^*flox/flox*^ females [64] to generate *Nodal* conditional mutants. *Notch3*^*Gt(PST033)Byg*^ (abbreviated *Notch3*^*lacZ*^, [65]) were maintained in a BALB/c background. Both male and female embryos were collected and used randomly for experiments, except for RNA sequencing, in which only male embryos were used to reduce variability. Embryonic day (E) 0.5 was defined as noon on the day of vaginal plug detection. Embryonic stages were determined based on the morphology of the heart according to [17] and [12]. Somite numbers were evaluated from brightfield images; samples with less than 18 somites were excluded from heart looping quantifications. All embryos were genotyped by PCR, using primers listed in S3 Table. Animals were housed in individually ventilated cages containing tube shelters and nesting material, maintained at 21 °C and 50% humidity, under a 12 h light/dark cycle, with food and water ad libitum, in the Laboratory of Animal Experimentation and Transgenesis of the SFR Necker, Imagine Campus, Paris. Animal procedures were approved by the ethical committees of the Institut Pasteur, Université Paris Cité and the French Ministry of Research (#18049-201707201335745 v9 and dha230022).

### Method details

#### RNA isolation and sequencing.

Paired left and right heart fields of E8.5f embryos were micro-dissected, from below the headfolds to the end of the second somite [19], after removal of the heart and the back, and bisected at the midline. The tissue was flash frozen in liquid nitrogen. All samples were collected within 1 h 30 min of sacrificing the mother. Total RNA was extracted in TRIzol-Chloroform and purified using the RNeasy micro kit (QIAGEN) including DNAse treatment. RNA quality and quantity were measured on Fragment Analyzer (Agilent Technologies). All RQN were >9.7. The libraries were established using the Nugen Universal Plus mRNA-Seq kit, using 15 ng of total RNA per sample. The oriented cDNAs produced from the poly-A+ fraction were PCR amplified (15–18 cycles). An equimolar pool of the final indexed RNA-Seq libraries was sequenced on an Illumina NovaSeq6000, with paired-end reads of 130 bases and a mean sequencing depth of 37.15 million reads per sample.

#### Embryo dissection.

Embryos were dissected in 1xDPBS and fixed in 4% paraformaldehyde either for 6 h at room temperature or 24 h at 4 °C. Yolk sac or tail pieces were collected for genotyping. For embryos dissected at E9.5, hearts were arrested in diastole by treatment with cold 250 mM KCl for 5 min. Fixed embryos were gradually dehydrated into methanol and stored at −20 °C. For pups collected at P0, they were euthanised, immerged in a cardioplegia solution (110 mM NaCl, 16 mM KCl, 16 mM MgCl_2_, 1.5 mM Cacl_2_, 10 mM NaHCO_3_) for 5 min and fixed in 4% PFA 24 h at 4 °C.

#### RNA in situ hybridization (ISH).

ISH was performed whole mount as in [12]. *Wnt11* and *Bmp2* antisense riboprobes were transcribed from plasmids. Signals were detected by alkaline phosphatase-conjugated anti-DIG antibodies (1/2,000), which were revealed with the BM purple (magenta) substrate. The samples were washed in 1× DPBS, post-fixed and imaged by HREM.

Wholemount RNAscope ISH were performed using the Mutliplex Fluorescent v2 Assay (Biotechne) and the protocol of [66]. *Notch3* (425171-C1, 425171-C2, 425171-C3) *Notch1* (404641-C2), *Notch2* (425161-C3), *Nodal* (436321-C1) and *Mab21l2* (456901) probes were used. Amplification steps were performed using the TSA cyanine5 and cyanine3 amplification kit. Hoechst (1/1,000) was used as a nuclear counterstain. Samples were then transferred in R2 CUBIC clearing reagents and embedded in R2 reagent containing agarose [67]. Multi-channel 16-bit images were acquired with a Z.1 lightsheet microscope and a 20×/1.0 objective. Specificity of the *Notch3* probe was controlled in mutants (S5 Fig) and comparatively to *Notch* paralogues (S3 Fig).

#### β-galactosidase staining.

*Notch3*^*LacZ/LacZ*^ embryos were collected at E9.5, fixed in 4% PFA, 5 mG EGTA, 2 mM MgCl_2_ for 10 min. They were permeabilised in 0.2% NP40, 2 mM MgCl_2_, 0.1% sodium deoxycholate 30 min and stained wholemount 45 min in Sgal solution (0.5 mg/ml Sgal, 0.3 mg/ml NBT in permeabilisation buffer) at 37 °C. Brightfield images were acquired with a Zeiss AxioCamICc5 Camera and a Zeiss StereoDiscovery V20 stereomicroscope with a Plan Apo 1.0× objective..

#### Reverse transcription quantitative polymerase chain reaction (RT-qPCR).

Reverse transcription was performed on RNA isolated from entire (left and right) micro-dissected heart fields using a Reverse Transcription kit (QuantiTect, Qiagen). Quantitative PCR was carried out using the ViiA7 real-time PCR system. mRNA expression levels were measured relatively to *Polr2b* and normalized with a reference cDNA sample, taken as a pool of 7 whole embryos at stage E8.5c-g, using the standard ΔΔCt method. Primers are listed in S3 Table.

#### High resolution episcopic microscopy (HREM).

E9.5 embryos or P0 hearts were imaged in 3D by HREM after embedding in methacrylate resin (JB4) containing eosin and acridine orange as contrast agents [12]. One or two channel images of the surface of the resin block were acquired using the optical high-resolution episcopic microscope and a 1× Apo objective repeatedly after removal of on average 1.75 or 2.63 µm thick sections: the tissue architecture was imaged with a GFP filter and the staining of β-galactosidase precipitates with an RFP filter. The datasets comprise 742–1869 images of 0.90–4.48 µm resolution in *x* and *y* depending on the stage. Occasionally, one slice may be lost or overexposed, creating a minor variation in the overall 3D stack. Icy [68] and Fiji softwares were used to format the datasets. 3D reconstructions and analysis were performed using Imaris. Figure panels correspond to optical sections in the plan which is most appropriate, i.e., not necessarily in the plan of imaging.

#### Micro-computed tomography.

The thoracic skin was removed to improve penetration of the contrast agent; the left arm was removed as a landmark of the fetus left side. Samples were stained in 100% lugol over 72 h (Desgrange, 2019). 3D images of the thorax and abdomen were acquired on a Micro-Computed Tomography Quantum FX (Perkin Elmer), within a field of exposer of 10 mm diameter. The dataset comprises 512 images of 20 × 20 × 20 µm (*x*–*y*–*z*) resolution for each sample. 3D reconstruction and analysis were performed with the Imaris software. Figure panels correspond to optical sections in the plan which is the most appropriate, i.e., not necessarily in the plan of imaging.

### Quantification and statistical analysis

#### Bioinformatics analyses of bulk RNA sequences.

FASTQ files were mapped to the ENSEMBL Mouse GRCm38/mm10 reference using Hisat2 and counted by featureCounts from the Subread R package. Due to a high number of duplicates (on average 89%), duplicates were excluded. Flags were computed from counts normalized to the mean coverage. All normalized counts in DESeq2 <20 were considered as background (flag 0) and ≥20 as signal (flag =  1). P50 lists used for the statistical analysis gather genes showing flag = 1 for at least half of the samples. Differential gene expression analysis was performed using three independent methods (DESeq2, edgeR and LimmaVoom) followed by thresholds on absolute fold change ≥1.2 and *p*-value ≤0.05, leading to 597 genes (S1 Table). Differential gene expression with LimmaVoom is plotted, and grouped in three categories depending on p-value and corrected *p*-value (Benjamini–Hochberg procedure) (S4 Table). Normalized read counts below 5.6 were considered as background, based on known markers included or excluded from the micro-dissected tissue (S1B–S1D Fig). For each differential analysis method, functional analyses were carried out using the QIAGEN Ingenuity Pathway analysis (QIAGEN, https://digitalinsights.qiagen.com/IPA) [69] on the list of differentially expressed genes, using delta between conditions and *p*-value of differential analysis. Transcriptomic data have been deposited in NCBI Gene Expression Omnibus (GEO) with the accession number GSE237126.

#### Bioinformatics analyses of published single cell RNA sequences.

Data from single cardiac wild-type cells at E8.5 [18] were downloaded from https://crukci.shinyapps.io/heartAtlas/ analyzed using scran [70] and visualized using Seurat v3.1.2 [71]. Data from single cardiac wild-type cells at E11.5-P9 [72] were downloaded from https://www.ncbi.nlm.nih.gov/geo/query/acc.cgi?acc=GSE193346, and visualized similarly.

#### Quantification of RNAscope ISH signal.

3D images were analyzed using Imaris. For quantification of left–right asymmetry in the second heart field, the second heart field was manually segmented in the lateral plate mesoderm layer, using headfolds and the second somite pair as cranial and caudal boundaries, respectively, and separated into left and right following the midline (using neural tube closure as a landmark). Both Hoechst signal and the signal of the gene of interest were extracted. To account for potential biases between the left and right during imaging, the spot detector function was used on the Hoechst channel to generate a normalization score between the two sides, using as a threshold a coverage of 50% of spots on each side. To calculate the left/right ratio of the gene of interest, its number of spots were measured on the left and right sides, normalized to Hoechst signal and to the volume of the segmented region.

#### Quantification of the geometry of the heart tube at E9.5.

HREM images were used to segment the different compartments of the heart tube in Imaris. Eight landmarks along the tube were extracted and used for quantifications as described previously [12]: one at the exit of the outflow tract, one at the sulcus between the two ventricles, and one at the bifurcation of the two atria (taken as the venous pole). The five other landmarks are centroids of cardiac regions: outflow tract, right ventricle, left ventricle, atrioventricular canal and left atrium, and right atrium, segmented according to the expression patterns of *Wnt11* and *Bmp2* and anatomical landmarks such as cushion boundaries or the interventricular or interatrial sulcus. Heart shapes were aligned in 3D using an in-house MATLAB code so that the *Z*-axis corresponds to the notochord axis and the x-axis to a perpendicular dorso-ventral axis. The orientation of the axis between the left and right ventricles, the distance of the venous pole to the notochord and the tube length were measured as in [17].

#### Phenotyping of congenital heart defects.

Hearts of *Notch3* mouse mutants were phenotyped at P0 in 3D images acquired by Micro-CT and HREM, based on the segmental approach [73] and International Paediatric and Congenital Cardiac Code and International Classification of Diseases (IPCCC ICD)-11 clinical code.

#### Human *NOTCH3* variants.

Variants were filtered in a cohort of 3,907 cases with congenital heart defects, reflecting a subset of a published cohort, for which data was shareable ([74], Ethics Approval EA2/131/10). Variant consequences were considered based on the *NOTCH3* transcript NM_000435.3. Filter criteria included an ultrarare population frequency (minimum allelic count (MAC) ≤  2 in population datasets (gnomAD V3, UK-biobank, internal control samples)). Variants were furthermore required to result in a protein truncation (stop gain, frameshift or canonical splicing variants expected to disrupt the transcript) or to have a severe altering effect. Severity of altering variants was assessed using a REVEL score ≥0.932 or CADD_phred ≥28.1 as suggested by Pejaver and colleagues [75]. Additional *NOTCH3* variants were obtained from ClinVar (https://simple-clinvar.broadinstitute.org, accessed September 26, 2024). Additional (likely) pathogenic variants associated with congenital heart defects (*n* = 303, S5 Table [74]) or heterotaxy (*n* = 566, S6 Table [9,10,62,76–78]) were filtered.

#### Statistical analyses.

Statistical tests and *p*-values are described in figure legends and S1 Data. Group allocation was based on PCR genotyping. All sample numbers (*n*) indicated in the text refer to biological replicates, i.e., different embryos or different cells. Investigators were blinded to allocation during imaging and phenotypic analysis, but not during quantifications. Pairwise Mann–Whitney–Wilcoxon tests were used to compare experimental groups, with a Benjamini–Hochberg correction applied on *p*-values when more than two populations are compared. A chi-squared test was used to compare percentage distributions, or a chi-squared test with Yates’ continuity correction for comparison with an expected value. Tests were performed with R and Excel. Embryos which had been damaged during experiments were excluded for quantification.

## Supporting information

S1 Fig Related to Fig 1. Transcriptomic approach and co-expression of *Nodal* and *Notch3.* (**A**) Brightfield images of wild-type embryos used for RNA sequencing at E8.5f. In the left panel, an outline of the dissected areas is shown. The identification number of embryos is given. (**B**) Normalized read counts of genes used to validate the threshold of expression in the transcriptomic analysis. The osteocyte gene *Dmp1*, inner ear marker *Oc90*, neuronal marker *Neurod1* are used as negative controls and *Mmp9* as a positive control, lowly expressed in left heart progenitors. Whisker plots show the median, 25th- and 75th quartiles (boxes), and the extreme data points (whiskers). (**C**) Normalized read counts of genes used as markers to control sample micro-dissection. *En2, Wnt8b* are anterior markers, *Hoxa5, Hoxb6* posterior markers, *Rfx4*, *Tfap2b* back markers, *Isl1, Six2, Fgf8* second heart field markers, *Tbx5, Mab21l2* juxta-cardiac field (JCF) markers and *Myh7b, Myoz1, Tnnt1* cardiomyocyte markers. The dotted line indicates the threshold of background expression. (**D**) Normalized read counts of genes used as markers to validate the left–right dissection of samples. NODAL targets *Lefty2* and *Pitx2,* as well as *Six2* label the left side. *Nodal* is turned off at E8.5f. *p-value between the left and right sides <0.05, ***Benjamini–Hochberg corrected *p*-value <0.00001 (LimmaVoom, *n* = 4). (**E**) Violin plot of *Dtx4* expression in single cells at E8.5 from [18], clustered as annotated (*n* = 89 Ec2, 59 Me2, 713 Me3, 221 Me4, 355 Me5, 65 Me6, 514 Me7). Dots are normalized reads per cell. (**F**) Violin plot of *Notch3* expression in single *Nodal*-negative (*n* = 1,559) and *Nodal*-positive (*n* = 309) cells of cardiac clusters (Me3–7) of [18] at E8.5 (stage 1 to Late Heart Tube, wild-type embryos). Seventy-five percent (234/309) of *Nodal*-positive cells also express *Notch3*. (G-H) Transverse section of the left lateral plate mesoderm, labeled by double wholemount RNAscope ISH of *Nodal* (red) and *Notch3* (white). The region of *Notch3* and *Nodal* co-expression is outlined in yellow (*n* = 3). LPM, lateral plate mesoderm; NT, neural tube. See also S1 Data for the underlying data.
(TIFF)

S2 Fig Related to Fig 1. Asymmetric expression of *Notch3* in the node. (**A**) Expression of *Notch3* (white) detected by whole mount RNAscope ISH in a E8.5b wild-type embryo, seen in a ventral view. Segmentation of the node is shown, bisected along the midline (dotted line), to quantify gene expression in the left (white) or right (yellow) node. Expression of *Notch3* within the segmented node is extracted in the right panel. (**B**) Quantification of *Notch3* asymmetric expression in the node at E8.5b-d. ***p-value < 0.001 to compare *Notch3* ratio with a symmetry hypothesis (Log2 ratio = 0) (Pairwise Mann–Whitney Wilcoxon tests, *n* = 15). Means and standard deviations are shown. L, left; R, right. See also S1 Data for the underlying data.
(TIFF)

S3 Fig Related to Fig 1. Comparative expression pattern of *Notch* paralogues. (**A**) Normalized read counts of genes encoding NOTCH receptors in the left (blue) and right (red) heart field at E8.5f. The dotted line indicates the threshold of background expression. *p-value < 0.05 (LimmaVoom, *n* = 4). Whisker plots show the median, 25th- and 75th quartiles (boxes), and the extreme data points (whiskers). (**B**) Relative *Notch1* (white) and *Notch3* (magenta) expression detected by double whole-mount RNAscope ISH at E8.5d, and shown in a front view (b1), and transverse sections (b2–b3), at the levels indicated in b1 (*n* = 4). (**C**) Relative *Notch2* (white) and *Notch3* (magenta) expression at E8.5d-e, shown in a front view (c1), and transverse sections (c2–c3), at the levels indicated in c1 (*n* = 5). (**D**) Violin plots of *Notch1–4* expression levels in single cells at E8.5 from [18], at stage 1 to LHF and clustered as annotated (*n* = 35 Me2, *n* = 627 Me3, *n* = 169 Me4, *n* = 287 Me5, *n* = 63 Me6, *n* = 386 Me7). (**E**) Co-expression of *Notch2* (white) and the juxta-cardiac field (JCF, red dotted outline) marker *Mab21l2* (magenta) at stage E8.5d (*n* = 8), and shown in a front view (a1) and transverse section (a2, at the level indicated in a1). Expression of *Notch3* within the segmented JCF is extracted in the right panel. The midline is indicated by a yellow dotted line. DA, dorsal aorta; Ec, endocardium; FP, floor plate; L, left; R, right; SHF, second heart field (green dotted outline); So, somite. See also S1 Data for the underlying data.
(TIFF)

S4 Fig Related to Fig 2. Normal *Nodal* expression in *Notch3* mutants. (**A**, **B**) Whole mount RNAscope ISH of *Nodal* in *Notch3^−^^/^^−^* mutant embryos at E8.5c in anterior (A) and posterior (B) frontal views. The midline of the embryo is indicated by a yellow dotted line. A transverse section at the level of the node (see b1) is shown in b2. Filled and empty arrowheads point to high and absent expression, respectively. L, left; LPM, lateral plate mesoderm; No, node; R, right.
(TIFF)

S5 Fig Related to Fig 3. *Notch3* inactivation in mutants. (**A**) Schema of *Notch3* alleles in wild types and *Notch3^−^^/^^−^* mutants, indicating the localization of exons and primers used for reverse transcription quantitative polymerase chain reaction (RT-qPCR). (**B**) Relative expression of *Notch3* in micro-dissected heart fields of littermate wild types (*n* = 5 E8.5e, 3 E8.5g), *Notch3*^*+/−*^ (*n* = 6 E8.5e, 6 E8.5g) and *Notch3*^*−/−*^ (*n* = 6 E8.5e, 4 E8.5g) embryos, quantified by RT-qPCR using the indicated primer pairs and normalized to wild types. **p-value < 0.01, ***p-value < 0.001 (Pairwise Mann–Whitney Wilcoxon tests with Benjamini–Hochberg correction). (**C**, **D**) *Notch3* (white) expression by whole mount RNAscope ISH in E8.5h control *Notch3*^*+/−*^ embryos (C), compared to mutant *Notch3*^*−/−*^ (D), shown in frontal views. Filled and empty arrowheads point to high and low expression, respectively. L, left; LPM, lateral plate mesoderm; NT, neural tube; R, right. See also S1 Data for the underlying data.
(TIFF)

S6 Fig Related to Fig 4. Laterality of visceral organs in *Notch3* mutants and fetal *Notch3* expression. (**A**, **B**) Coronal sections of control *Notch3^*+*^^/^^−^* (A) and mutant *Notch3^−^^/^^−^* (B) neonates at P0, imaged by micro-computed tomography. (a1–b1) The heart apex situs (yellow arrowhead) is in levocardia. Right (RV) and left (LV) ventricles are correctly lateralized. (a2–b2) The situs of bronchi, of lung lobes, the position and shape of the stomach (St), spleen (S), liver (Li) and colon (Col) are all normal. LB, left bronchus; LLL, left lung lobe; RB, right bronchus; RLL, right lung lobe. (**C**) Violin plot of *Notch3* expression after heart looping, in single cardiac cell transcriptomic between E11.5 and P9 (from [72]), clustered as annotated (*n* = 5,422 atrial cardiomyocytes, 10,493 ventricular cardiomyocytes, 2,361 endocardium, 1,176 vascular endocardium, 1,012 epicardium, 3,309 fibroblast like cells, 182 smooth muscle cells, 349 pericytes, 1,032 macrophages, 33 B cells, 42 T cells, 17 dendritic cells, 23 natural killer cells, 22 neutrophils, 244 blood cells). Dots are normalized reads per cell. See also S1 Data for the underlying data.
(TIF)

S1 Video Related to Fig 1. Co-expression of *Notch3* and *Nodal.* *Notch3* (white) and *Nodal* (magenta) double whole-mount RNAscope ISH in a wild-type E8.5d embryo imaged in 3D by lightsheet microscopy.
(MP4)

S1 Table Related to Fig 1C. List of 597 differentially expressed genes used for Ingenuity Pathway analysis, based on a fold change ≥1.2 and *p*-value ≤0.05 with either the LimmaVoom, DESeq2 or edgeR methods. Flags indicate the number of samples in which normalized read counts are ≥20 (*n* = 4 embryos). Lf, left heart field at E8.5f; Rf, right heart field at E8.5f.
(XLSX)

S2 Table List of ultrarare and likely pathogenic *NOTCH3* variants in patients with congenital heart defects. Variants are classified according to the ACMG guidelines [55]. Variants in orange are associated with congenital heart defects also found in patients with Lateral Meningocele syndrome [49], whereas variants in blue are associated with congenital heart defects also found in patients with heterotaxy [3]. SNV, single nucleotide variant; VUS, variant of uncertain significance.
(XLSX)

S3 Table List of primers used for genotyping and reverse transcription quantitative polymerase chain reaction (RT-qPCR). (XLSX)

S4 Table Related to Fig 1B. List of 466 differentially expressed genes with LimmaVoom. The normalized number of reads are shown. Genes have been filtered for a *p*-value < 0.05. Individual samples are L29_3, L43_5, L46_8, L49_3. These data were used in the Volcano plot or to plot expression levels. Full data are available in GSE237126. L, left; R, right.
(XLSX)

S5 Table List of CHD-related genes used to screen additional pathogenic variants in S2 Table. (XLSX)

S6 Table List of plausibly associated heterotaxy genes used to screen additional pathogenic variants in S2 Table. (XLSX)

S1 Data Numerical data used to generate figure graphs along with statistical tests. (XLSX)

## Abbreviations

ACMG: American College of Medical Genetics and Genomics
Ao: aorta
AVC: atrioventricular canal
BH: Benjamini–Hochberg
BMP: bone morphogenetic protein
E: Embryonic Day
HREM: high resolution episcopic microscopy
HT: Heart Tube
ISH: in situ hybridization
IPCCC ICD: International Paediatric and Congenital Cardiac Code and International Classification of Diseases
L: left
LA: left atrium
LPM: lateral plate mesoderm
LV: left ventricle
MAC: minimum allelic count
Myo: myocardium
No: node
n.s.: non-significant
NT: neural tube
P: postnatal day
R: right
RA: right atrium
RT-qPCR: reverse transcription quantitative polymerase chain reaction
RV: right ventricle
SHF: second heart field
TGFβ: Transforming Growth Factor-β
VSD: ventricular septal defect
