# Supplementary figures and images for: Notch3 is an asymmetric gene and a modifier of heart looping defects in Nodal mouse mutants
Source: PLoS Biol. 2025 Mar 31;23(3):e3002598. doi: 10.1371/journal.pbio.3002598 (PMC12135939; doi:10.1371/journal.pbio.3002598)

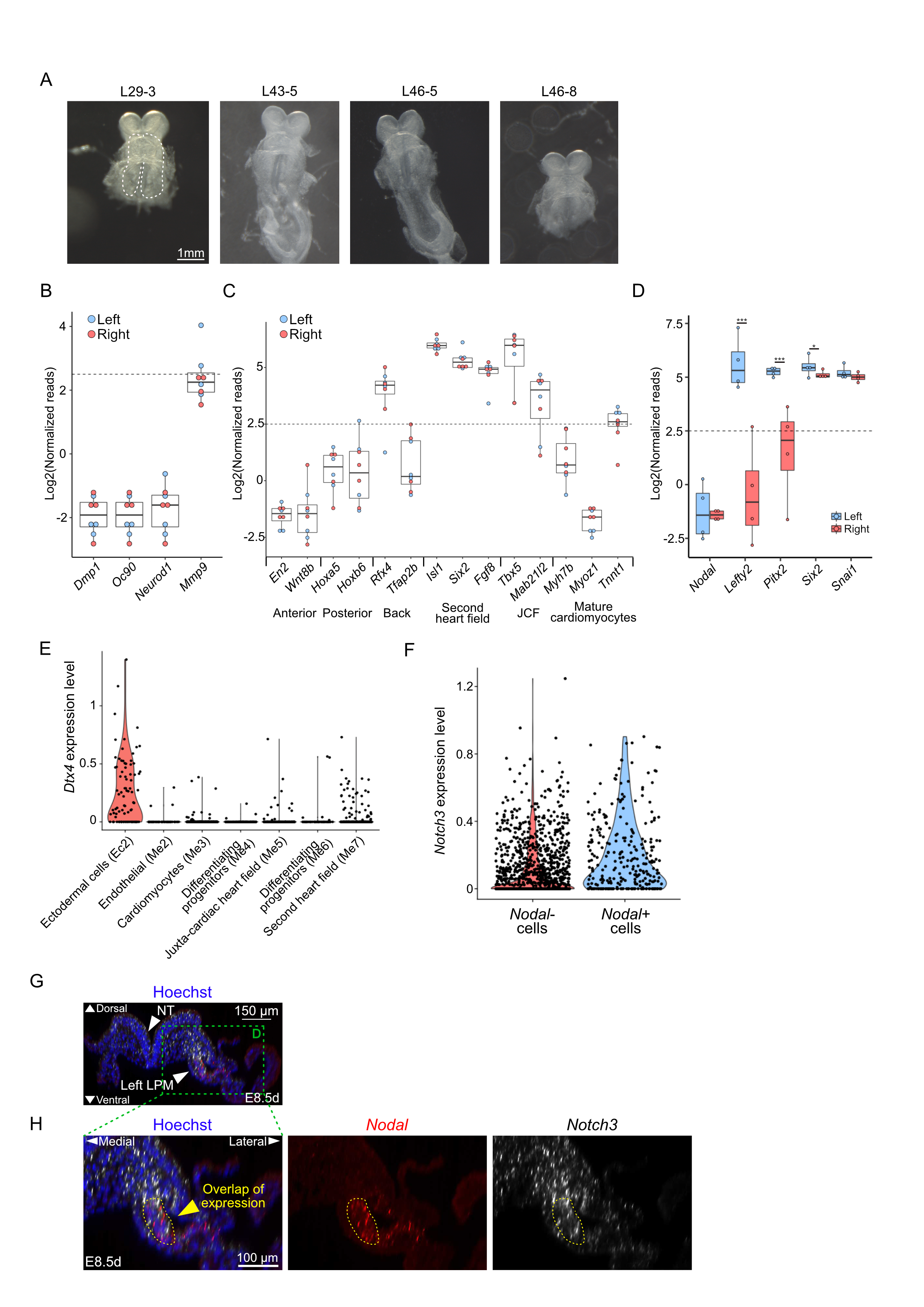

Supplement: S1 Fig — (A) Brightfield images of wild-type embryos used for RNA sequencing at E8.5f. In the left panel, an outline of the dissected areas is shown. The identification number of embryos is given. (B) Normalized read counts of genes used to validate the threshold of expression in the transcriptomic analysis. The osteocyte gene Dmp1, inner ear marker Oc90, neuronal marker Neurod1 are used as negative controls and Mmp9 as a positive control, lowly expressed in left heart progenitors. Whisker plots show the median, 25th- and 75th quartiles (boxes), and the extreme data points (whiskers). (C) Normalized read counts of genes used as markers to control sample micro-dissection. En2, Wnt8b are anterior markers, Hoxa5, Hoxb6 posterior markers, Rfx4, Tfap2b back markers, Isl1, Six2, Fgf8 second heart field markers, Tbx5, Mab21l2 juxta-cardiac field (JCF) markers and Myh7b, Myoz1, Tnnt1 cardiomyocyte markers. The dotted line indicates the threshold of background expression. (D) Normalized read counts of genes used as markers to validate the left–right dissection of samples. NODAL targets Lefty2 and Pitx2, as well as Six2 label the left side. Nodal is turned off at E8.5f. *p-value between the left and right sides <0.05, ***Benjamini–Hochberg corrected p-value <0.00001 (LimmaVoom, n = 4). (E) Violin plot of Dtx4 expression in single cells at E8.5 from [18], clustered as annotated (n = 89 Ec2, 59 Me2, 713 Me3, 221 Me4, 355 Me5, 65 Me6, 514 Me7). Dots are normalized reads per cell. (F) Violin plot of Notch3 expression in single Nodal-negative (n = 1,559) and Nodal-positive (n = 309) cells of cardiac clusters (Me3–7) of [18] at E8.5 (stage 1 to Late Heart Tube, wild-type embryos). Seventy-five percent (234/309) of Nodal-positive cells also express Notch3. (G-H) Transverse section of the left lateral plate mesoderm, labeled by double wholemount RNAscope ISH of Nodal (red) and Notch3 (white). The region of Notch3 and Nodal co-expression is outlined in yellow (n = 3). LPM, lateral plate mesode [file pbio.3002598.s001.tiff]

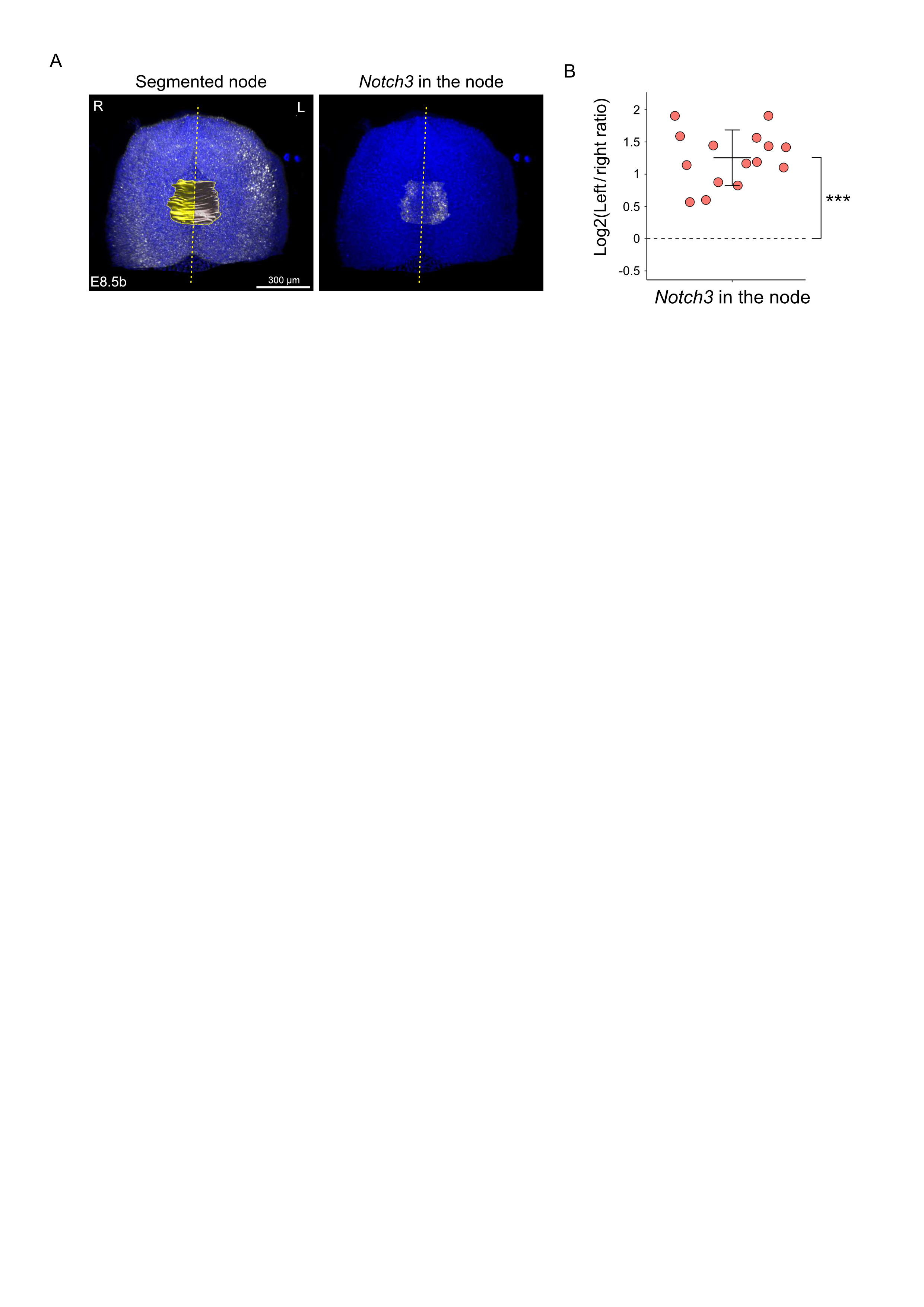

Supplement: S2 Fig — (A) Expression of Notch3 (white) detected by whole mount RNAscope ISH in a E8.5b wild-type embryo, seen in a ventral view. Segmentation of the node is shown, bisected along the midline (dotted line), to quantify gene expression in the left (white) or right (yellow) node. Expression of Notch3 within the segmented node is extracted in the right panel. (B) Quantification of Notch3 asymmetric expression in the node at E8.5b-d. ***p-value < 0.001 to compare Notch3 ratio with a symmetry hypothesis (Log2 ratio = 0) (Pairwise Mann–Whitney Wilcoxon tests, n = 15). Means and standard deviations are shown. L, left; R, right. See also S1 Data for the underlying data. (TIFF) [file pbio.3002598.s002.tiff]

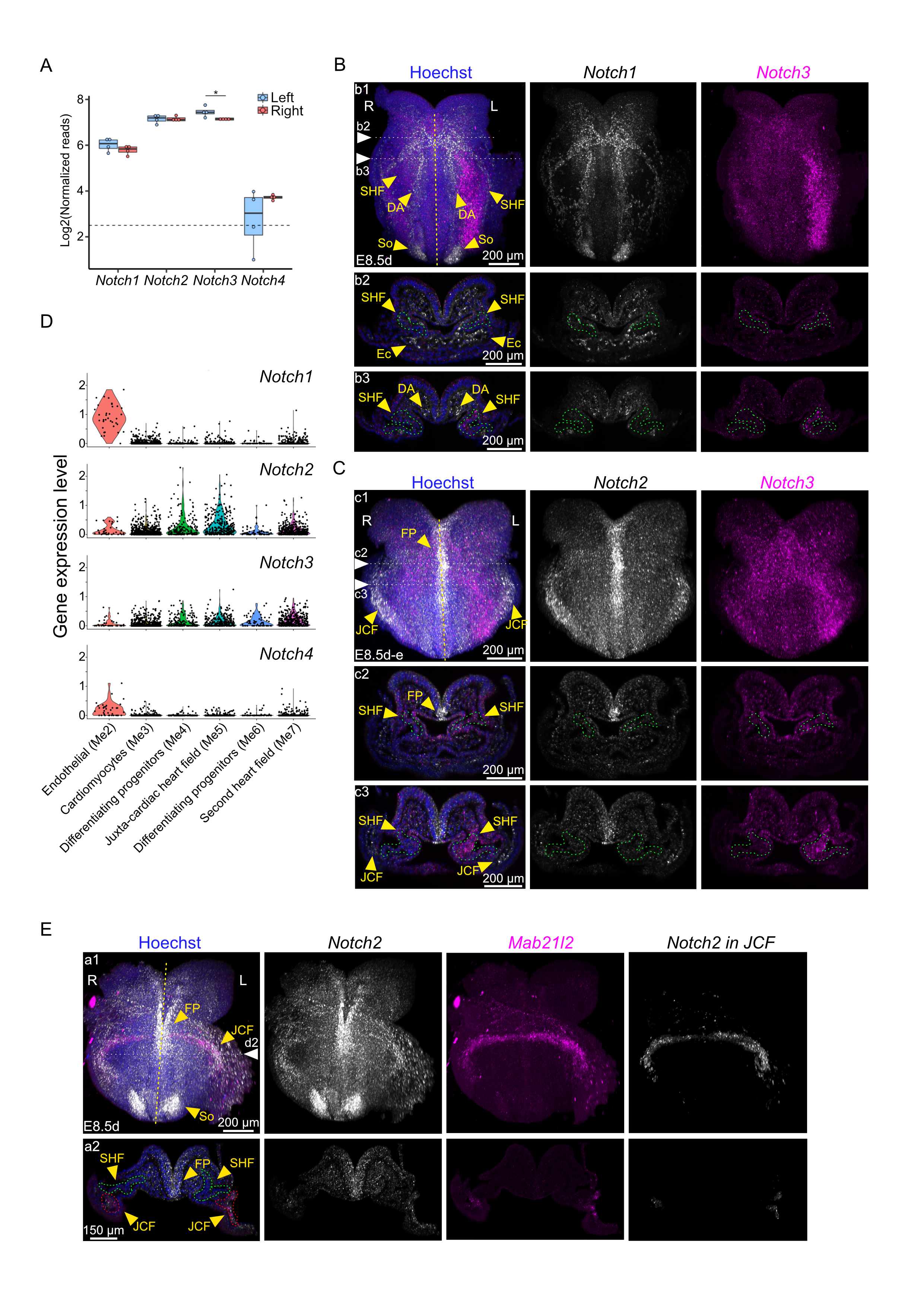

Supplement: S3 Fig — (A) Normalized read counts of genes encoding NOTCH receptors in the left (blue) and right (red) heart field at E8.5f. The dotted line indicates the threshold of background expression. *p-value < 0.05 (LimmaVoom, n = 4). Whisker plots show the median, 25th- and 75th quartiles (boxes), and the extreme data points (whiskers). (B) Relative Notch1 (white) and Notch3 (magenta) expression detected by double whole-mount RNAscope ISH at E8.5d, and shown in a front view (b1), and transverse sections (b2–b3), at the levels indicated in b1 (n = 4). (C) Relative Notch2 (white) and Notch3 (magenta) expression at E8.5d-e, shown in a front view (c1), and transverse sections (c2–c3), at the levels indicated in c1 (n = 5). (D) Violin plots of Notch1–4 expression levels in single cells at E8.5 from [18], at stage 1 to LHF and clustered as annotated (n = 35 Me2, n = 627 Me3, n = 169 Me4, n = 287 Me5, n = 63 Me6, n = 386 Me7). (E) Co-expression of Notch2 (white) and the juxta-cardiac field (JCF, red dotted outline) marker Mab21l2 (magenta) at stage E8.5d (n = 8), and shown in a front view (a1) and transverse section (a2, at the level indicated in a1). Expression of Notch3 within the segmented JCF is extracted in the right panel. The midline is indicated by a yellow dotted line. DA, dorsal aorta; Ec, endocardium; FP, floor plate; L, left; R, right; SHF, second heart field (green dotted outline); So, somite. See also S1 Data for the underlying data. (TIFF) [file pbio.3002598.s003.tiff]

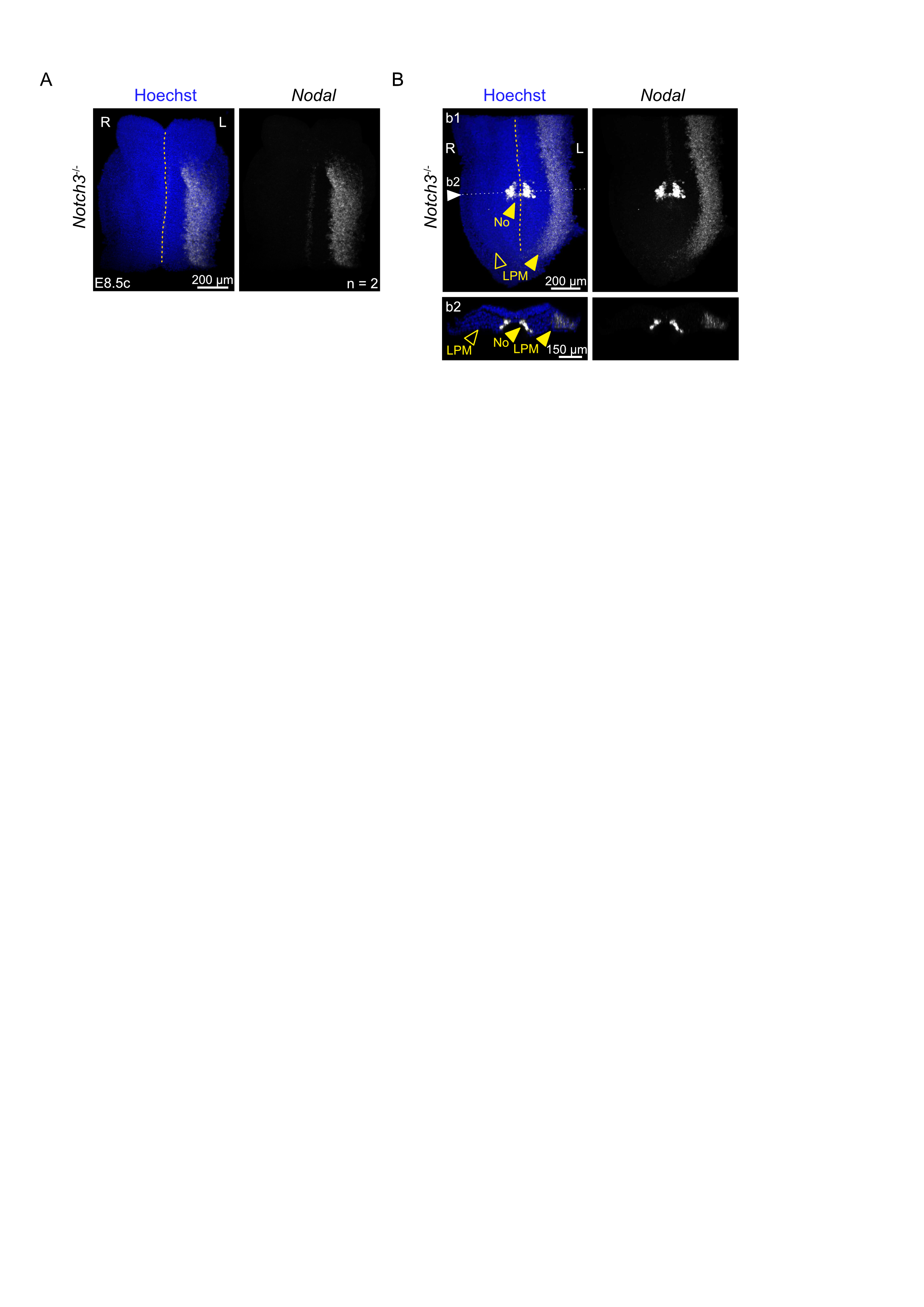

Supplement: S4 Fig — (A, B) Whole mount RNAscope ISH of Nodal in Notch3−/− mutant embryos at E8.5c in anterior (A) and posterior (B) frontal views. The midline of the embryo is indicated by a yellow dotted line. A transverse section at the level of the node (see b1) is shown in b2. Filled and empty arrowheads point to high and absent expression, respectively. L, left; LPM, lateral plate mesoderm; No, node; R, right. (TIFF) [file pbio.3002598.s004.tiff]

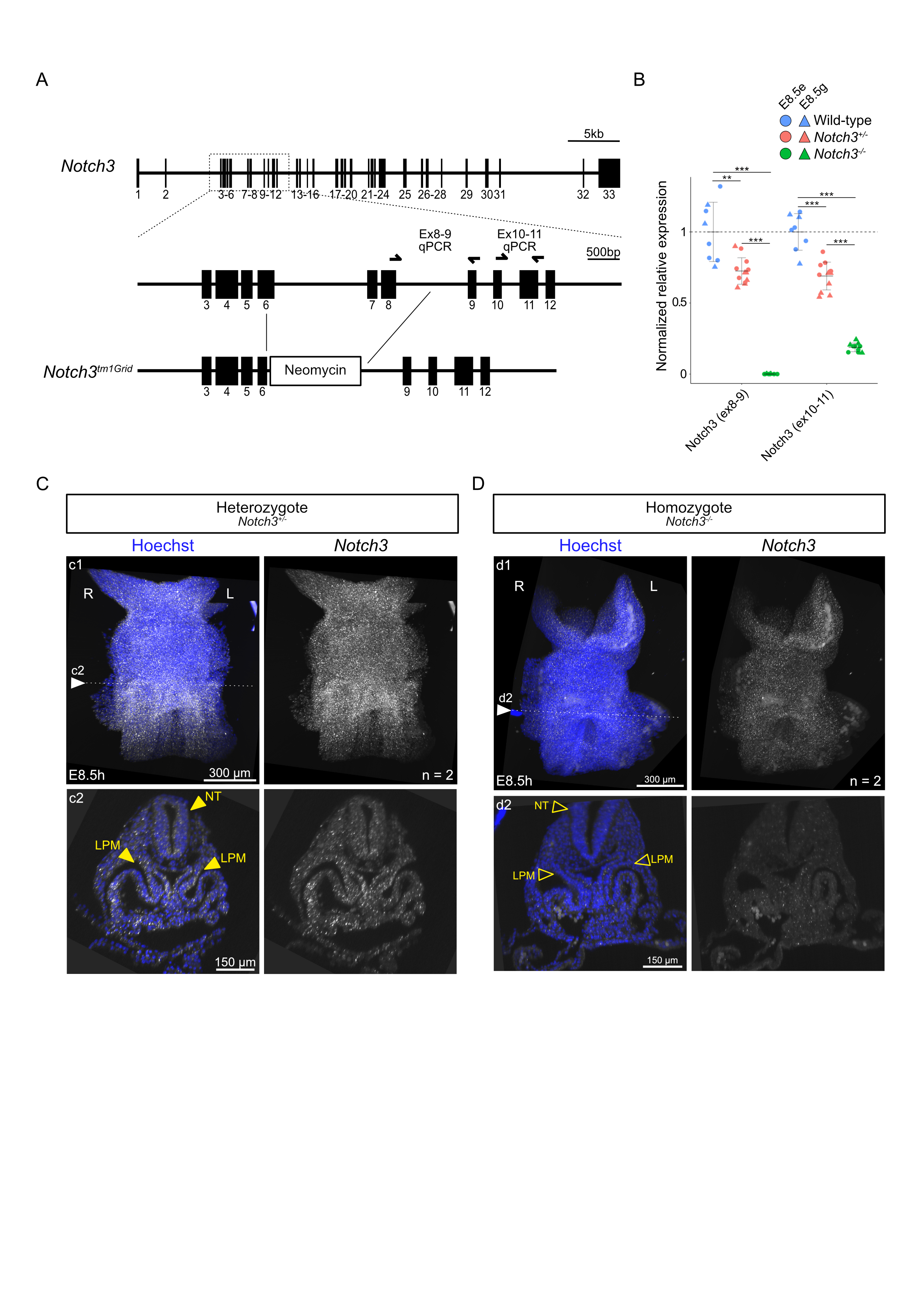

Supplement: S5 Fig — (A) Schema of Notch3 alleles in wild types and Notch3−/− mutants, indicating the localization of exons and primers used for reverse transcription quantitative polymerase chain reaction (RT-qPCR). (B) Relative expression of Notch3 in micro-dissected heart fields of littermate wild types (n = 5 E8.5e, 3 E8.5g), Notch3+/− (n = 6 E8.5e, 6 E8.5g) and Notch3−/− (n = 6 E8.5e, 4 E8.5g) embryos, quantified by RT-qPCR using the indicated primer pairs and normalized to wild types. **p-value < 0.01, ***p-value < 0.001 (Pairwise Mann–Whitney Wilcoxon tests with Benjamini–Hochberg correction). (C, D) Notch3 (white) expression by whole mount RNAscope ISH in E8.5h control Notch3+/− embryos (C), compared to mutant Notch3−/− (D), shown in frontal views. Filled and empty arrowheads point to high and low expression, respectively. L, left; LPM, lateral plate mesoderm; NT, neural tube; R, right. See also S1 Data for the underlying data. (TIFF) [file pbio.3002598.s005.tiff]

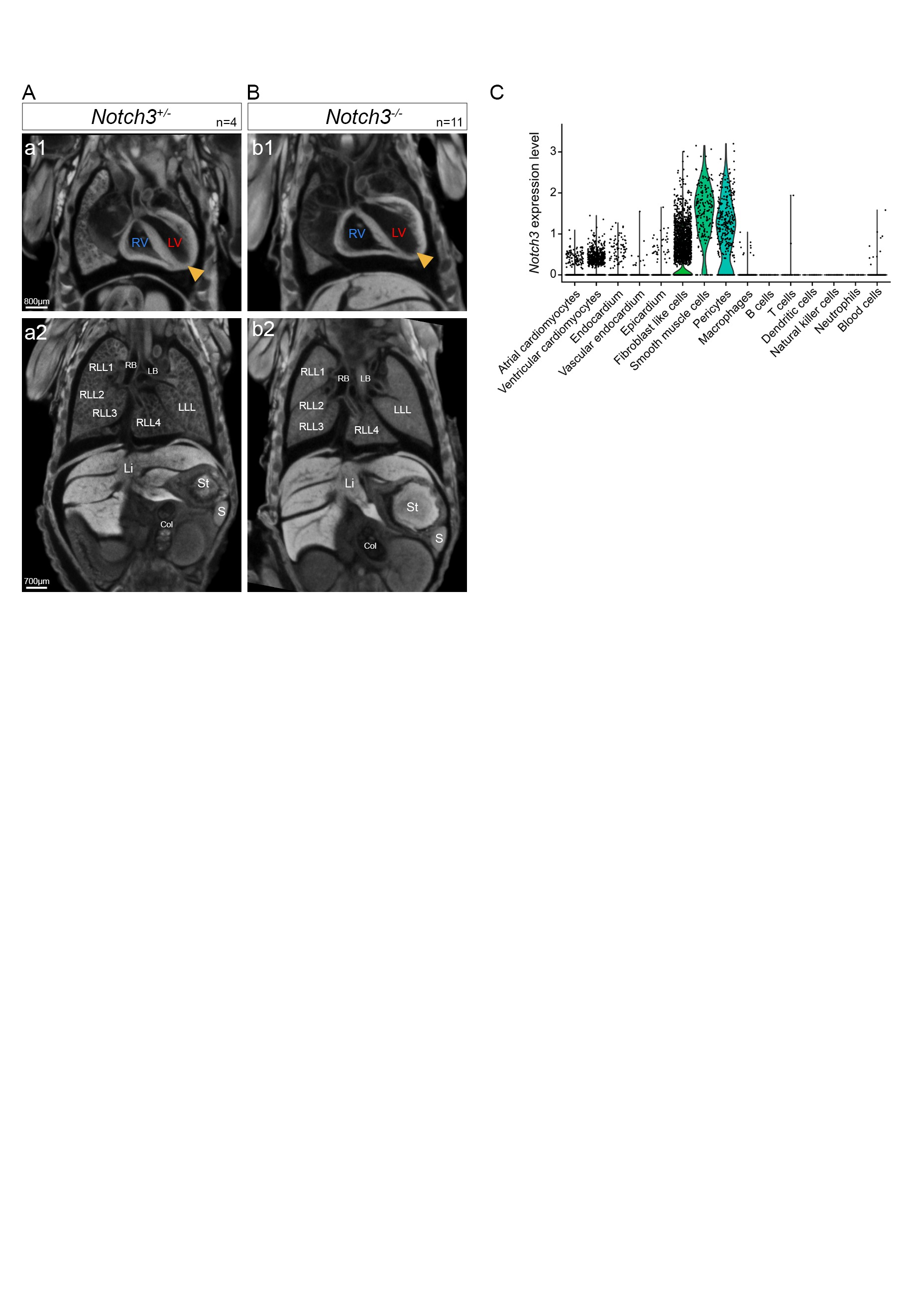

Supplement: S6 Fig — (A, B) Coronal sections of control Notch3+/− (A) and mutant Notch3−/− (B) neonates at P0, imaged by micro-computed tomography. (a1–b1) The heart apex situs (yellow arrowhead) is in levocardia. Right (RV) and left (LV) ventricles are correctly lateralized. (a2–b2) The situs of bronchi, of lung lobes, the position and shape of the stomach (St), spleen (S), liver (Li) and colon (Col) are all normal. LB, left bronchus; LLL, left lung lobe; RB, right bronchus; RLL, right lung lobe. (C) Violin plot of Notch3 expression after heart looping, in single cardiac cell transcriptomic between E11.5 and P9 (from [72]), clustered as annotated (n = 5,422 atrial cardiomyocytes, 10,493 ventricular cardiomyocytes, 2,361 endocardium, 1,176 vascular endocardium, 1,012 epicardium, 3,309 fibroblast like cells, 182 smooth muscle cells, 349 pericytes, 1,032 macrophages, 33 B cells, 42 T cells, 17 dendritic cells, 23 natural killer cells, 22 neutrophils, 244 blood cells). Dots are normalized reads per cell. See also S1 Data for the underlying data. (TIF) [file pbio.3002598.s006.tif]
